# Machine learning based predictors for COVID-19 disease severity

**DOI:** 10.1038/s41598-021-83967-7

**Published:** 2021-02-25

**Authors:** Dhruv Patel, Vikram Kher, Bhushan Desai, Xiaomeng Lei, Steven Cen, Neha Nanda, Ali Gholamrezanezhad, Vinay Duddalwar, Bino Varghese, Assad A Oberai

**Affiliations:** 1 Viterbi School of Engineering, University of Southern California, Los Angeles, CA USA; 2 Keck School of Medicine, University of Southern California, Los Angeles, CA USA

**Keywords:** Prognosis, Machine learning, Outcomes research

## Abstract

Predictors of the need for intensive care and mechanical ventilation can help healthcare systems in planning for surge capacity for COVID-19. We used socio-demographic data, clinical data, and blood panel profile data at the time of initial presentation to develop machine learning algorithms for predicting the need for intensive care and mechanical ventilation. Among the algorithms considered, the Random Forest classifier performed the best with $\text {AUC} = 0.80$ for predicting ICU need and $\text {AUC} = 0.82$ for predicting the need for mechanical ventilation. We also determined the most influential features in making this prediction, and concluded that all three categories of data are important. We determined the relative importance of blood panel profile data and noted that the AUC dropped by 0.12 units when this data was not included, thus indicating that it provided valuable information in predicting disease severity. Finally, we generated RF predictors with a reduced set of five features that retained the performance of the predictors trained on all features. These predictors, which rely only on quantitative data, are less prone to errors and subjectivity.

## Introduction

The current coronavirus disease 2019 (COVID-19) pandemic has strained healthcare delivery models across the world. In the US there are over 8 million cases and 5.4% have required hospitalization. Of the hospitalized patients, to date, 20% have required care in the intensive care unit (ICU)^1^. Based on current projections, by January 1st 2021 the number of ICU beds needed for COVID patients will exceed the available ICU beds by 10.6%^2,3^. With this challenge in supply of ICU beds, states and counties have created detailed surge plans to ensure timely care of critically ill patients suffering with COVID-19. In order to sustain healthcare delivery through this pandemic, it is imperative to adopt a proactive approach towards utilization of healthcare resources like ICU beds and ventilators. Given the urgency for resource allocation and optimization, we sought to identify patient-level clinical characteristics at the time of admission to predict the need for ICU care and mechanical ventilation in COVID-19 patients.

Several studies have reported predictors for the severity of COVID-19 that are trained on data acquired at or around the time of admission^4–7^. The study described in this manuscript differs from these in several significant ways. First, instead of applying a single predictive model, we assess the performance of a cohort of models and then select the one that performs the best. Second, we do not include any imaging data and rely only on socio-demographic data, data acquired from a physical exam, and lab marker data obtained from a blood draw. This combination may be relevant to facilities in under-resourced scenarios where rapid imaging is not available. Third, we evaluate the relative benefit in predictive accuracy that is obtained from the lab-marker data alone, and conclude that it is significant. Fourth, we also consider a reduced model with only five features as input, and report good predictive performance for our model. This simplified model is easy to use, and only contains quantitative features thereby making it less prone to error and subjectivity. Finally, in training our model we consider data from Los Angeles county, while other studies are based on populations in other world regions. This is relevant since the outcome for COVID-19 are known to be dependent on demographics.

## Methods

Data for this study was extracted from an Institutional Review Board (IRB) approved COVID-19 REDCap^8^ repository. Informed consent for the repository was waived by the USC IRB consistent with §45 CFR 46.116(f). The study was conducted in accordance with USC policies, IRB policies, and federal regulations. Subjects’ privacy and confidentiality were protected according to applicable HIPAA, and USC IRB policies and procedures. The repository contained demographic, clinical, and laboratory data for all COVID-19 positive patients seen at the Keck Medical Center of USC, Verdugo Hills Hospital, and Los Angeles County + USC Medical Center. Repository data elements include data from three categories: (a) socio-demographic data including age, sex, travel, contact history, and co-morbidities; (b) presenting clinical data gleaned from symptoms and the results of an initial physical examination including fever, dyspnea, respiratory rate, and blood oxygen saturation (SpO_2_); (c) blood panel profile including RT-PCR, InterLeukin-6, d-Dimer, complete blood count, lipase, and C-reactive protein (CRP). They also include the outcome data, namely, the need for ICU admission and mechanical ventilation. A description of all the input features, their type, and their median, minimum and maximum values is presented in Tables 1, 2, 3, 4 and 5.

**Table 1:** Socio-demographic features used as input.

Socio-demographic numerical features	Median	Min	Max
Age (years)	53	12	93
BMI ($\text {kg}/\text {m}^{2}$)	29	0	84.05

**Table 2:** Socio-demographic features used as input.

Socio-demographic categorical features	Distribution
Sex	Male (58.02%), Female (41.98%)
Pregnant	Yes (2.83%), No (93.87%), Unsure (3.30%)
Race	American Indian or Alaska Native (0%), Asian (3.77%), Black or African American (5.66%), Native Hawaiian or Other Pacific Islander (0%), White (22.64%), Other (67.92%)
Ethnicity	Hispanic/Latino (59.43%), Non-Hispanic/Non-Latino (30.66%), Unknown (9.91%)
International travel	Yes (7.08%), No (92.92%)
Primary contact	Yes (17.45%), No (40.09%), Unsure (42.45%)
Secondary contact	Yes (10.38%), No (42.92%), Unsure (46.70%)
Other contact	Yes (9.91%), No (41.04%), Unsure (49.06%)
Work contact	Yes (10.84%), No (89.16%)

**Table 3:** Input features from presenting clinical data and the results of an initial physical examination.

Clinical numerical features	Median	Min	Max
Days since symptoms presented (days)	5	1	29
Systolic blood pressure (mmHg)	129.5	54	228
Diastolic blood pressure (mmHg)	75.5	34	116
Heart rate (bpm)	106	53	156
Respiratory rate (br/min)	20	12	48
Body temperature (°C)	37.11	35	39.7
SpO_2_ (%)	95	48	100

**Table 4:** Input features from presenting clinical data and the results of an initial physical examination.

Clinical categorical features	Distribution
Immuno-compromised	Yes (11.79%), No (88.21%)
Cardiac history	Yes (7.55%), No (92.45%)
Diabetes mellitus	Yes (31.13%), No (68.87%)
COPD	Yes (1.89%), No (98.11%)
Asthma	Yes (6.60%), No (93.40%)
Interstitial lung disease	Yes (0.47%), No (99.53%)
Obesity	Yes (33.96%), No (66.04%)
Auto-immune disease	Yes (5.66%), No (94.34%)
Hypertension	Yes (38.21%), No (61.79%)
Other morbidity	Yes (48.11%), No (51.89%)
Fever	Yes (53.77%), No (46.23%)
Chills	Yes (40.57%), No (59.43%)
Shortness of breath or dyspnea	Yes (60.85%), No (39.15%)
Chest pain	Yes (16.98%), No (83.02%)
Cough	Yes (74.06%), No (25.94%)
Loss of smell	Yes (3.77%), No (96.23%)
Loss of taste	Yes (11.79%), No (88.21%)
Body ache/myalgia	Yes (36.79%), No (63.21%)
Fatigue	Yes (25.94%), No (74.06%)
Throat pain	Yes (15.09%), No (84.91%)
Abdominal pain	Yes (10.85%), No (89.15%)
Diarrhea	Yes (17.92%), No (82.08%)
Influenza like illness symptoms	Yes (37.26%), No (62.74%)
Other symptom	Yes (60.85%), No (39.15%)
General appearance	Normal (61.17%), Abnormal (29.61%), Not done (9.22%)
Head	Normal (76.62%), Abnormal (2.99%), Not done (20.40%)
Eyes	Normal (78.00%), Abnormal (1.00%), Not done (21.00%)
Ears	Normal (73.47%), Abnormal (0%), Not done (26.53%)
Nose	Normal (73.87%), Abnormal (1.51%), Not done (24.62%)
Throat	Normal (75.62%), Abnormal (2.99%), Not done (21.39%)
Chest and lungs	Normal (42.31%), Abnormal (57.21%), Not done (0.48%)
Heart	Normal (68.27%), Abnormal (24.52%), Not done (7.21%)
Abdomen	Normal (77.45%), Abnormal (7.84%), Not done (14.71%)
Extremities	Normal (77.45%), Abnormal (6.86%), Not done (15.69%)
Nervous system	Normal (78.71%), Abnormal (11.39%), Not done (9.90%)
Skin	Normal (71.78%), Abnormal (3.47%), Not done (24.75%)

**Table 5:** Input features from blood panel profile.

Blood panel features	Median	Min	Max
Glucose (mg/dL)	131	53	575
Calcium (mg/dL)	8.7	6.7	11.2
Albumin (g/dL)	3.9	0	4.7
Total protein (g/dL)	7.1	0	9.3
Sodium (mmol/L)	136	124	154
Potassium (mmol/L)	4.1	2.7	6.3
Bicarbonate (total CO_2_) (mmol/L)	23	11	37
Chloride (mmol/L)	98	84	114
Blood urea nitrogen (BUN) (mg/dL)	13	0.56	137
Creatinine (mg/dL)	0.84	0.37	17.59
Alkaline phosphatase (ALP) (U/L)	80	29	417
Alanine amino transferase (ALT/SGPT) (U/L)	35.5	5	247
Aspartate amino transferase (AST/SGOT) (U/L)	47	13	355
Bilirubin (mg/dL)	0.5	0.2	20.5
C-reactive protein (CRP) (mg/L)	91.7	0.6	470.8
d-dimer (mcg/mL FEU)	0.81	0.14	20
Procalcitonin (ng/mL)	0.18	0.02	31.9

The study cohort comprised of 212 patients (123 males, 89 females) with an average age of 53 years (13–92 years), of which 74 required intensive care at some point during their stay, and 47 required mechanical ventilation. We note that only data obtained at the time of initial presentation, with 24 hours of initial presentation, was included as input to the predictive models, and the need for ICU admission and mechanical ventilation at any time during hospitalization were selected as outcomes.

Features with more than 30% missing data were excluded from the analysis. In the retained features, missing data was imputed using an iterative imputation method. In this method the feature to be imputed is treated as a function of a subset of other highly-correlated features and missing values are obtained using regression^9^. This subset of features is then iterated over to arrive at the final estimate. As part of this strategy, in order to prevent data leakage, only the training samples were used to develop regression models for imputation.

The retained features were used to compute the correlation of the outcome with input features. Thereafter, data was split into training (60%), and testing sets (20%). Fivefold cross-validation was performed using the training set to train the supervised learning models and tune their hyperparameters (random forest, multilayer perceptron, support vector machines, gradient boosting, extra tree classifier, adaboost). Among all these algorithms the Random Forest^10^ (RF) classifier was found to be the most accurate and was considered for further analysis.

The tuned RF model was applied to testing data to compute the probability of ICU admission and mechanical ventilation. This was repeated with five different folds, yielding predicted probabilities for 212 subjects generated by five distinct RF models. These were used to generate an ROC curve and compute the area under the curve (AUC). The relative importance of the input features was evaluated by computing their Gini importance.

The analysis describe above was first performed with input data from all categories, that is, socio-demographic data, presenting clinical data, and blood panel profile data. Thereafter, the blood panel profile data was excluded and the analysis was performed once again. This second analysis was done to assess the relative importance of the blood panel data in predicting the outcomes.

## Results

**Figure 1:**
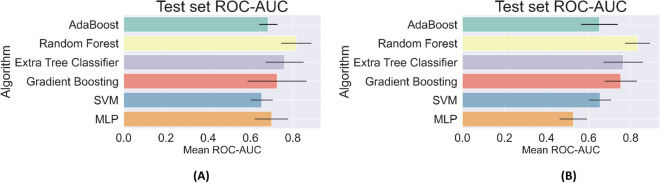
Area under the curve (AUC) for the classifiers considered in the study for predicting the need for ICU (**A**) and mechanical ventilation (**B**).

In Fig. 1, we have plotted the AUC values for predicting the need for ICU and mechanical ventilation for all the algorithms considered in this study. From this figure we observe that the algorithms based on decision trees, that is, Random Forest, Extra Tree Classifier, and Gradient Boosting tend to perform better. This is likely because the simpler algorithms like Support Vector Machines do not have sufficient capacity to capture the complexity in the prediction, while other algorithms like Multi-Layer Perceptrons (MLP) do not have sufficient data for efficient training. This leads to issues with robustness and over-fitting. Further, among the algorithms based on decision trees, the Random Forest (RF) classifier is the most accurate and was considered for further analysis.

For the RF predictor, we reported an AUC of 0.80, 95% CI (0.73–0.86) in predicting the need for ICU and an AUC of 0.83, 95% CI (0.76–0.90) for predicting the need for mechanical ventilation. At the optimal cut-point in the ROC curve^11^, the ICU predictor yields a Sensitivity of 0.73, Specificity of 0.74, a Positive Predictive Value (PPV) of 0.6 and a Negative Predictive Value (NPV) of 0.84, whereas the predictor for Mechanical Ventilation yields a Sensitivity of 0.72, Specificity of 0.73, a PPV of 0.44 and an NPV of 0.90 (see Table 6). These values demonstrate that we are able to accurately predict the need for intensive care and ventilation from data acquired at the time of admission. In terms of the AUC, the performance of the RF predictor is similar to results reported in studies from China^4^, New York^7^ and the Netherlands^5^ (AUC of 0.88, 0.8, and 0.77, respectively). We note that these studies differ from ours due to the regional differences in the population and the viral strain. Further, some these studies also included chest x-ray imaging features and tested a single type of ML algorithm (logistic regression or random forest). Deep learning models were also developed based on a cohort from China^6^, and these report an AUC 0.89 for a coarse measure of disease severity that clubs together patients receiving ICU care or mechanical ventilation, and those ultimately succumbing to the disease.

When only socio-demographic and presenting clinical data was used as input (lab markers were excluded), the AUC value for predicting ICU need dropped to 0.68, 95% CI (0.60–0.75), and that for predicting ventilation dropped to 0.70, 95% CI (0.61–0.79). The values of Sensitivity, Specificity, PPV and NPV at the optimal point also dropped by about 0.1 (see Table 6). This indicates that the lab marker data provides significant additional information and is important in improving the accuracy of these predictions. A recent comprehensive survey of laboratory markers concluded that many of the markers that are included in this study are correlated with COVID-19 severity and should therefore be used in models for predicting disease severity^12^. However, our results also indicate that it is possible to make moderately accurate predictions with only socio-demographic and presenting clinical data. This is particularly useful when quick decisions are required and the time or resources necessary for acquiring lab marker data are not available in a timely manner.

**Table 6:** Performance of Random Forest Predictors at the optimal operating point.

Model	Sensitivity	Specificity	PPV	NPV
ICU (w. lab markers)	0.73 (0.63, 0.83)	0.74 (0.67, 0.81)	0.60 (0.50, 0.70)	0.84 (0.77, 0.90)
ICU (no lab markers)	0.64 (0.53, 0.74)	0.64 (0.56, 0.72)	0.48 (0.39, 0.58)	0.77 (0.69, 0.84)
ICU (five features)	0.70 (0.60, 0.81)	0.70 (0.63, 0.78)	0.56 (0.46, 0.66)	0.82 (0.75, 0.88)
Ventilation (w. lab markers)	0.72 (0.60, 0.85)	0.73 (0.67, 0.80)	0.44 (0.33, 0.55)	0.90 (0.85, 0.95)
Ventilation (no lab markers)	0.60 (0.46, 0.74)	0.61 (0.53, 0.68)	0.30 (0.21, 0.39)	0.84 (0.77, 0.91)
Ventilation (five features)	0.74 (0.62, 0.87)	0.75 (0.68, 0.81)	0.45 (0.34, 0.57)	0.91 (0.86, 0.96)

We report Sensitivity, Specificity, Positive Predictive Value (PPV), and Negative Predictive Value (NPV). Numbers in parenthesis are the 95% confidence interval.

**Figure 2:**
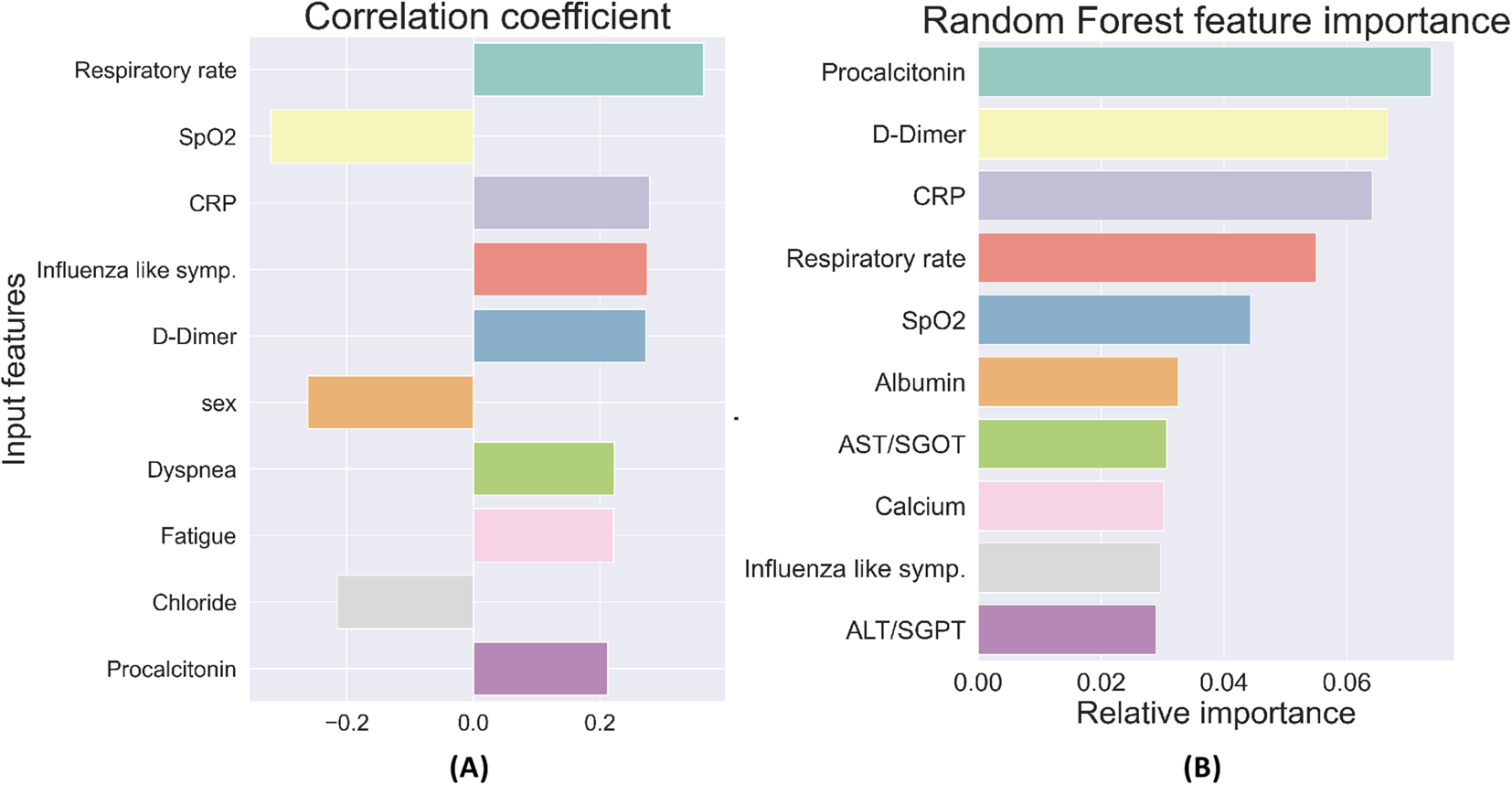
(**A**) Ten most highly correlated features with the need for ICU care. (**B**) Ten features with the highest relative importance for predicting the need for ICU care.

**Figure 3:**
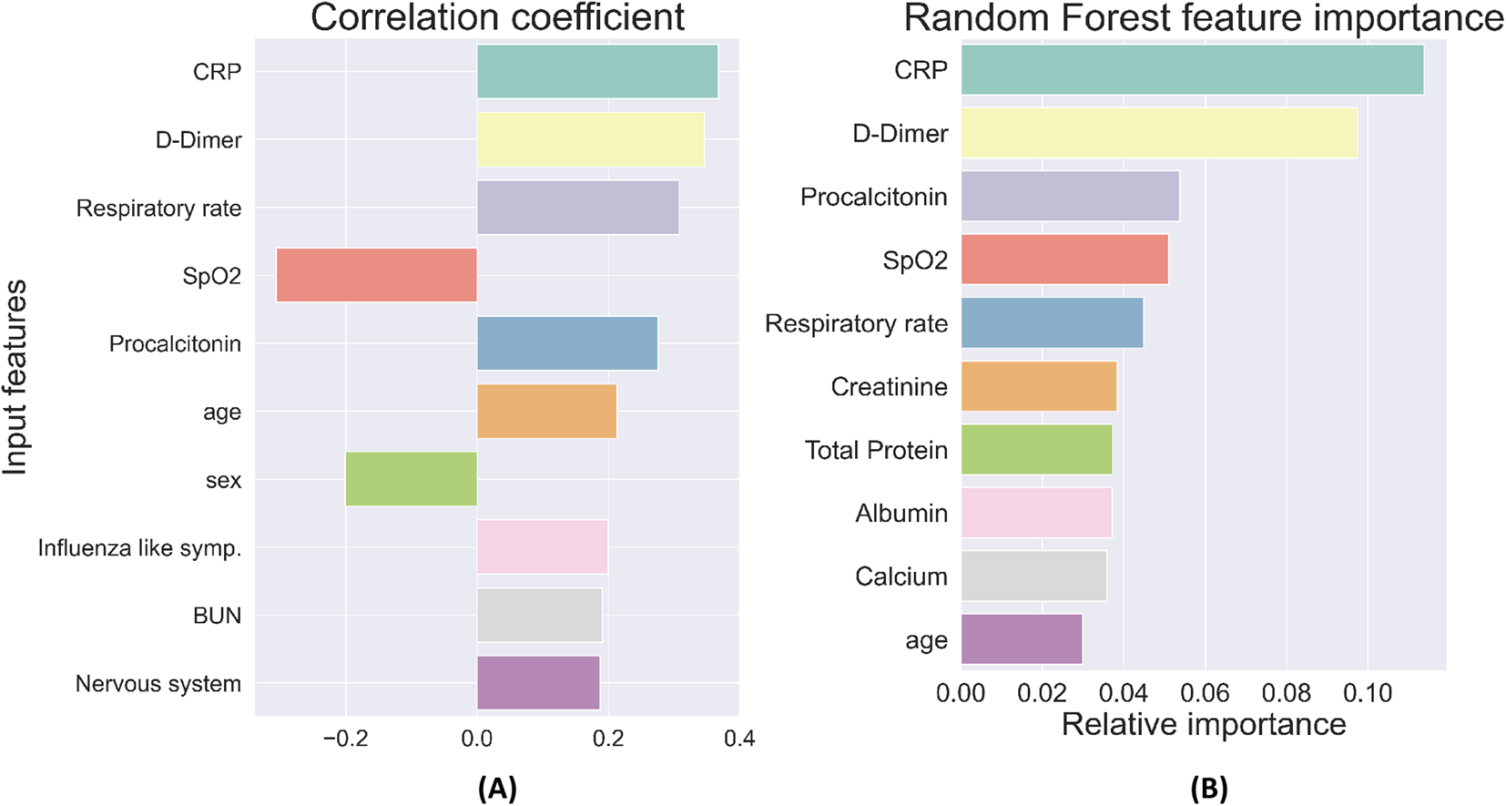
(**A**) Ten most highly correlated features with the need for mechanical ventilation. (**B**) Ten features with the highest relative importance for predicting the need for mechanical ventilation.

The top ten features with the strongest correlation to ICU admission are shown in Fig. 2A, and the most important features for the RF classifier for ICU need are shown in Fig. 2B. Similarly, the top ten features with the strongest correlation to the need for mechanical ventilation are shown in Fig. 3A, and the most important features for the RF classifier for mechanical ventilation need are shown in Fig. 3B.

Taken together, this set represents features that strongly influence the likelihood of ICU admission and mechanical ventilation. We note that they belong to all three categories—socio-demographic data, presenting clinical data, and blood panel profile data—showing that all these type of data are necessary in making an accurate assessment of disease severity. Several of these features have been implicated in determining the severity of COVID-19 by other researchers^7,13–19^; however, there are few studies that have considered them together and determined their relative importance.

Finally, we considered RF predictors that are trained only using the top five features for predicting ICU need. These are the values for CRP, d-Dimer, Procalcitonin, SpO_2_, and respiratory rate. Models based on this reduced set of features are easier to implement since they require less data. They are also more robust and not prone to subjective assessment since all these features are quantitative numbers that can be measured accurately. For the model designed to predict ICU need using these features we report an AUC of 0.79 (0.72, 0.85) and for the model designed to predict the need for mechanical ventilation we report an AUC of 0.83 (0.77, 0.9). Both these values are very close to the corresponding predictors that utilize all 72 features, thereby indicating not much accuracy is lost by employing the simpler, more robust models. The sensitivity, specificity, PPV and NPV values for these reduced models are reported in the third and sixth rows of Table 6, and these are also quite close to the corresponding models that utilize all 72 features.

**Figure 4:**
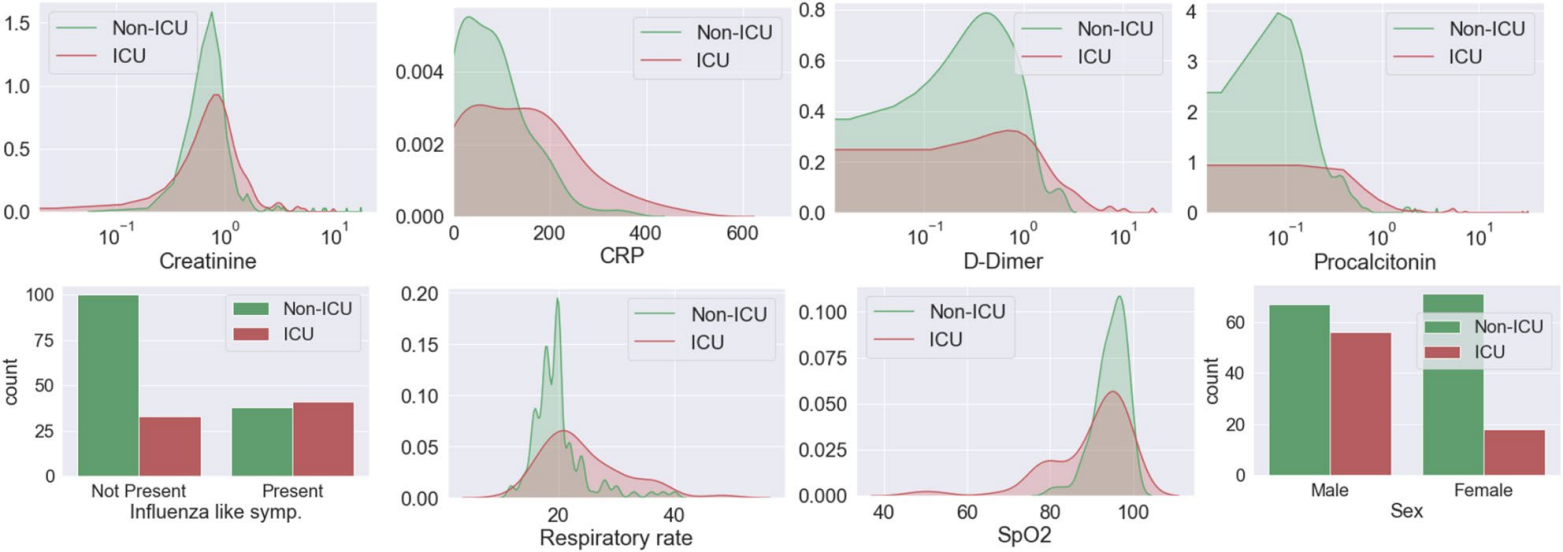
Distribution of (from top left to bottom right) Creatinine, C-reactive Protein (CRP), d-Dimer, Procalcitonin, influenza-like symptoms, respiratory rate, SpO_2_ level, and sex for patients admitted to ICU and those who are not.

In Fig. 4, we plot the distribution of some of the most important input features, including lab markers, presenting symptoms, and socio-demographic data for two sets of patients: those who require ICU care and whose who do not. We observe that the distribution of Creatinine (indicator of kidney function), C-reactive Protein (measure of inflammatory response), d-Dimer (measure of blood clot formation and breakdown), and Procalcitonin (elevated during infection and sepsis) among patients who require ICU care is spread over a larger range and has a higher average value. A similar trend is observed in the distribution for the respiratory rate. For SpO_2_ levels also we observe a distribution spread over a wider range for patients admitted to the ICU; however, in this case this group has a lower average value. We also note that the presence of the influenza-like symptoms roughly doubles the likelihood of requiring ICU care (from around 25% to 52%). Further, the percentage of males who are admitted to the ICU is much higher than the percentage of females (46% to 20%).

## Discussion

The results presented in this study demonstrate that data acquired at or around the time of admission of a COVID-19 patient to a care facility can be used to make an accurate assessment of their need for critical care and mechanical ventilation. Further, the important features in this data belong to three different sets, namely, socio-demographic data, presenting clinical data, and blood panel profile data. We report that in cases where the blood panel data is not available, useful prediction might still be made, albeit with some loss of accuracy. This would be relevant to situations where the time or resources to acquire this type of data are limited. Out of all the machine learning models considered in this study, we found the random forest to be most accurate and robust to data perturbation for both critical care and mechanical ventilation prediction. We also demonstrate that the values of just five features, namely, CRP, Procalcitonin, d-Dimer, SpO_2_, and respiratory rate, can be used to predict the need for critical care and mechanical ventilation with an accuracy that is comparable to using all 72 features. The list of important features identified in our study is also indicative of a disease that affects multiple systems in the body including the respiratory, the circulatory system, and the immune system.

## Data Availability

The datasets generated during and/or analysed during the current study are available from the corresponding author on reasonable request.
